# An Organ-on-a-Chip Modular Platform with Integrated Immunobiosensors for Monitoring the Extracellular Environment

**DOI:** 10.3390/mi16070740

**Published:** 2025-06-25

**Authors:** Anastasia Kanioura, Myrto Kyriaki Filippidou, Dimitra Tsounidi, Panagiota S. Petrou, Stavros Chatzandroulis, Angeliki Tserepi

**Affiliations:** 1 Institute of Nuclear & Radiological Sciences and Technology, Energy & Safety, NCSR “Demokritos”, Patriarchou Gregoriou E’ and 27 Neapoleos Str., Aghia Paraskevi, Attiki, 15341 Athens, Greece; nkanioura@ipta.demokritos.gr (A.K.); dtsounidi@rrp.demokritos.gr (D.T.); ypetrou@rrp.demokritos.gr (P.S.P.); 2 Institute of Nanoscience and Nanotechnology, NCSR “Demokritos”, Patriarchou Gregoriou E’ and 27 Neapoleos Str., 15341 Aghia Paraskevi, Greece; myrto50x@gmail.com (M.K.F.); s.chatzandroulis@inn.demokritos.gr (S.C.)

**Keywords:** organ-on-chip, biosensors, reduced graphene oxide (rGO) sensors, IL-6 detection, PCB biochips

## Abstract

OoC systems employing human cells mirror the functionality of human organs and faithfully simulate their physiological microfluidic environment. Despite the potential of OoC technology in emulating tissue complexity, a significant gap persists in the continuous real-time monitoring of cellular behaviors and their responses to external stimuli, arising from the lack of biosensors integrated onto OoC microfluidic platforms. Addressing this limitation constitutes the primary objective of this study. By developing and incorporating biosensors onto a modular integrated OoC platform, we aim to enable the monitoring of changes taking place in the cellular environment under various stimuli in real time. An in-series modular integration of a biosensor array into an OoC platform is demonstrated herein, along with its potential to sustain human cell proliferation and accommodate the detection of IL-6, as an example of a mediator protein secreted as part of the immune response to inflammation. The implementation of commercially fabricated PCB components also addresses the issue of cost efficiency and manufacturing scaling-up of sensor-integrated OoCs. This advancement will not only enhance the accuracy and reliability of preclinical studies, but also pave the way for improved drug development and disease treatment.

## 1. Introduction

The preclinical evaluation of drug candidates involves cell culture and animal studies, both of which exhibit limitations in mimicking the complex cellular microenvironment within the human body, thus impeding accurate prediction of human cellular responses [1]. The scientific community is now relying on organ-on-a-chip (OoC) technology as a promising route in preclinical drug evaluation, disease modeling, and personalized medicine. OoC systems utilize human cells to mimic the functionality of human organs and simulate their physiological microfluidic environment. Despite the potential of OoC technology in emulating tissue complexity, a significant gap persists in the continuous real-time monitoring of cellular behaviors and their responses to external stimuli. To address this challenge, organ-on-chips (OoCs) integrated with sensors have emerged as a promising solution [2] to monitor both the cell culture conditions and biological responses of the cultured cells.

Integrating in situ analysis methods on-chip enables improved time resolution, continuous measurements, and faster read-outs. The integration of biosensors in line within OoC systems has enabled the real-time monitoring and detection of cell-secreted soluble biomarkers, providing unprecedented insights into cellular responses and disease mechanisms. Integrated mechanical, electrical, electrochemical, and optical sensors have started to find use in organ-on-chip systems, as discussed in several recent reviews [3,4,5]. Notable examples include the liver-on-chip platform by Zhang et al. (2017), featuring multi-sensor (physical, biochemical, and optical) integration for the continuous monitoring of liver-specific proteins and drug metabolism [6]. Similarly, a heart-on-chip system by Park et al. (2019) incorporated fluorescence-based biosensors for the real-time detection of cardiac-specific proteins, facilitating the evaluation of drug-induced cardiotoxicity and the assessment of therapeutic interventions [7]. In a skeletal muscle-on-a-chip study by Ortega et al. (2019), the integration of amperometric ELISA allowed for the identification of interleukin-6 (IL-6) and tumor necrosis factor-alpha (TNF-α), providing a means for observing inflammation within muscle tissue due to exposure to electrical signals and lipopolysaccharide (LPS) stimuli [8]. In cases where highly multiplexed measurements of secreted proteins were needed [9], highly miniaturized microwell arrays and digital microfluidic chips were developed; however, detection was achieved off-chip, e.g., via image analysis of microwell array images [10] and LC-MS [11], respectively.

Interleukins are of particular interest as mediators secreted during the immune response in which the cells of our immune system communicate with each other, and are produced by many types of cells, including white blood cells. Interleukin-6 (IL-6), first identified in the mid-1980s, is a pleiotropic glycoprotein produced by a wide range of cells in response to a variety of stimuli, and regulates various events, such as the immune response, hematopoiesis, inflammation, cell division and differentiation, survival, and the apoptosis of cells [12,13]. In healthy adults, IL-6 plasma concentrations are <10 pg/mL, while its production increases sharply during acute inflammatory reactions associated with injury, stress, infection, brain death, and others, reaching several ng/mL in cases of autoimmune diseases and even concentrations at the μg/mL level during septic shock [14,15]. Additionally, elevated levels of IL-6 are strongly related to the growth, differentiation, and immigration of tumor cells, as well as with resistance to chemotherapy and radiotherapy, rendering IL-6 one of the most important cytokines in the tumor microenvironment [16]. In this context, IL-6 detection has been demonstrated in point-of-care (POC) platforms for early disease (e.g., sepsis, cancer) diagnosis and prognosis [17,18], as well as in OoC models for monitoring inflammatory processes including tumor progression and therapeutic resistance [19,20]. For the latter application, in particular, it has been documented in the literature that chronic inflammation, as evidenced by IL-6 secretion, in the tumor microenvironment facilitates tumor growth and induces resistance toward chemo- and radiotherapy. However, in most cases, IL-6 detection is performed off-chip, in collected OoC effluents at the chip outlet. Sensor-integrated OoC for the in-situ detection of IL-6 has been demonstrated only in one case, by means of SERS [21], for monitoring IL-6 secreted by LPS-stimulated A549 cells. In addition, two aspects that are important to consider when developing sensor-integrated OoCs is the possibility for production scale-up and the manufacturing cost associated with each chip; however, these issues are rarely addressed in the literature [3,22]. In fact, PCB-based platforms, as in [22], allow for the monitoring of physical parameters (e.g., temperature) but not biochemical ones (e.g., biomarkers), as in the work herein.

**Figure 1:**
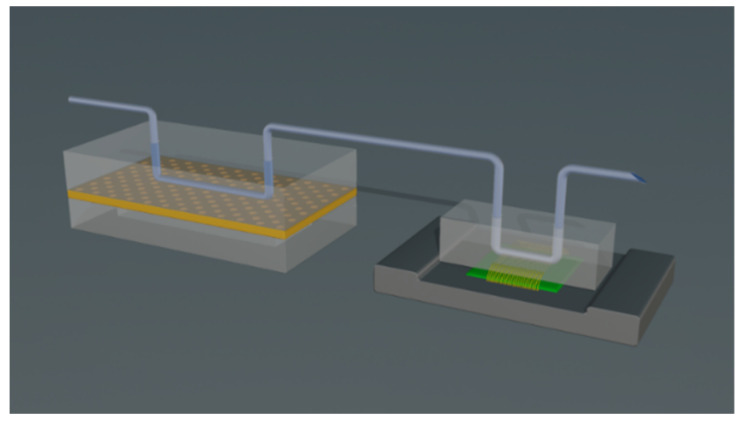
Modular OoC platform scheme integrated with biosensors.

In this work, a modular OoC platform incorporating an immunosensor and a Micro-fluidic Perfusion Chamber (MPC) for the real-time monitoring of the cellular microenvironment is presented. In particular, we propose a setup for the incorporation of a reduced graphene oxide (rGO) immunosensor array on commercially realized printed circuit boards (PCB), intended for IL-6 detection in an OoC during the culturing of breast cancer cell line MCF7, for which increased levels of IL-6 secretion have been associated with poor prognosis and resistance in therapy in breast cancer patients [23]. The development of such sensors addresses the current challenges of high manufacturing costs, production scale-up, and standardization in the OoCs field [3]. In addition, employing the soft lithography technique, we successfully fabricated a microfluidic perfusion chamber (MPC) [24] incorporating a microchannel and a medium reservoir, separated by a porous polyester membrane on which MCF7 cells were cultured. To preserve the integrity of the cultured MCF7 cells and prevent disruptions, the biosensor unit resides downstream the MPC in a modular integrated OoC platform (Figure 1), allowing the cell culture medium to flow through the cultured cells first and subsequently to the microchannel hosting the sensors array for the detection of cell-secreted analytes. This strategic arrangement can facilitate the direct and real-time detection of proteins, enabling precise monitoring and analysis of cellular responses in a continuous and dynamic manner. For the immunochemical detection of IL-6 with the rGO biosensors array, an indirect immobilization approach was followed involving the modification of the sensor with streptavidin to enable the binding of a biotinylated antibody specific for IL-6, aiming to avoid excessive loss of antibody binding capacity due to interaction with the sensor surface.

## 2. Materials and Methods

### 2.1. Materials

Polydimethylsiloxane (PDMS) elastomer (Sylgard 184 Silicone Elastomer Kit) was purchased from Dow Corning (Midland, MI, USA). Ordyl SY 300 was acquired from Resistechno (Elga Europe SLR, Milan, Italy). Transparent porous polyester membranes suitable for cell culture, ipCELLCULTURE™, were purchased from ip4it (Louvain-la-Neuve, Belgium), while graphene oxide (GO) solution “Single Layer Graphene Oxide Ethanol Dispersion” was obtained from ACS Material^®^ (Pasadena, CA, USA). Recombinant human procalcitonin and human interleukin-6 were purchased from CUSAg (Wuhan, China), and mouse monoclonal antibody against human IL-6 (code 2706) was purchased from Medix Biochemica (Espoo, Finland). Bovine serum albumin (BSA) was purchased from Acros Organics (Geel, Belgium), and ImmunoPure Streptavidin was obtained from Thermo Fisher Scientific (Waltham, MO, USA). MCF-7 cells were obtained from ATCC (Manassas, VA, USA). Dulbecco’s modified Eagle medium (DMEM), fetal bovine serum (FBS), L-glutamine, penicillin/streptomycin, and 0.05%/0.02% (*w*/*v*) trypsin-EDTA solution in PBS were obtained from Biowest (Nuaillé, France), while CellTracker Green was purchased from Thermo Fisher Scientific (Whaltham, MA, USA).

### 2.2. Fabrication of a Microfluidic Perfusion Chamber (MPC)

**Figure 2:**
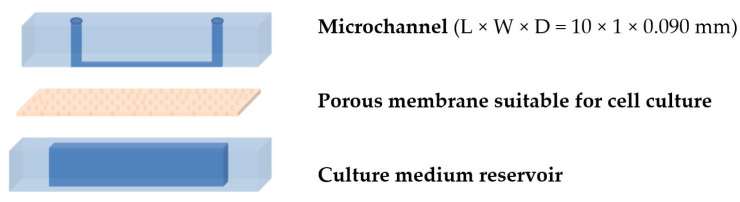
Schematic representation of the MPC platform. The nominal microchannel dimensions (length L, width W, and depth D) are indicated.

A 3D perfused microchannel, the structure of which (Figure 2) was selected from typical OoCs mimicking key functions of the human kidney proximal tubule [25,26], was fabricated from PDMS using replica molding. Each of its components was reproduced as the negative of a mold on which the PDMS prepolymer (a mixture of base and curing agent at a ratio of 10:1) was poured. As a mold for the culture medium reservoir, an unstructured aluminum container was implemented, while for the fabrication of the microchannel mold, a computer-aided design (FreeCAD 0.20) software program was used for designing the photolithographic mask of the microchannel (1 mm wide, 10 mm in length). The microchannel mold was created through negative tone photolithography of Ordyl (90-μm thick) dry film resist laminated on PCB substrates measuring 7 cm in length and 2 cm in width, thus enabling the patterning of two microchannels on each mold. Subsequently, the microchannel mold was affixed to the base of an aluminum container (73 mm in length, 22 mm in width, and 5 mm in height). Τhe molded PDMS was baked at 100 °C for 60 min, peeled off from the mold, and cut to uniform dimensions of 3 cm in length and 1.5 cm in width. At the microchannel layer, two holes were punched as the inlet and outlet at the microchannel ends, where plastic tubing would be attached before the injection of the fluids. The bottom reservoir was fabricated by removing a rectangular section (1.1 mm in width, 1.1 cm in length) from the unstructured PDMS slab. To complete the 3D assembly, a transparent porous polyester membrane suitable for cell culture was interposed between the microchannel and reservoir PDMS pieces, which were subsequently bonded together using plasma treatment (Femto Science Inc., Hwaseong, Republic of Korea).

### 2.3. Cell Culture

MCF7 cells were grown in DMEM enriched with 10% (*v*/*v*) fetal bovine serum, 2 mM L-glutamine, and 1% penicillin/streptomycin, and maintained at a constant temperature of 37 °C in an environment with high humidity and 5% CO_2_ until they reached 70–80% confluence. Afterwards, the cells were incubated with DMEM containing 1 μM Cell Tracker Green for 30min. Then, the cells were treated with trypsin-EDTA, and upon detachment from the culture flasks, the cells were counted, centrifuged, and condensed at a concentration of 1 × 10^6^ cells/mL.

The OoC platform was sterilized through exposure to ultraviolet (UVC) light in a laminar flow hood for 20 min, prior to the introduction of the cell suspension into the OoC microchannel followed by culture for 24 and 72 h at 37 °C in an environment with high humidity and 5% CO_2_. An Axioskop 2 Plus microscope, Carl Zeiss, Hamburg, Germany, was used to examine the cells’ distribution and development, while photos were taken using a 3.3 RTV CCD camera (QImaging, Surrey, BC, Canada) using the program Image Pro Plus v6 (Media Cybernetics, Inc., Rockville, MD, USA).

### 2.4. Sensor Preparation and Interleukin-6 Detection Protocol

**Figure 3:**
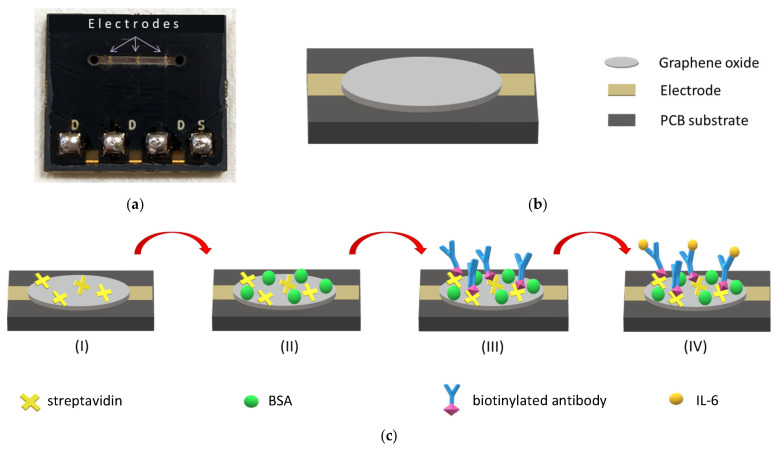
(**a**) Biosensor chip realized on PCB; (**b**) schematic illustration of the modification with graphene oxide sensor; and (**c**) schematic representation of the sensor functionalization and IL-6 detection, including (I) immobilization of streptavidin onto the chip surface, (II) blocking with bovine serum albumin, (III) incubation with biotinylated antibody against IL-6, and (IV) incubation with IL-6.

The sensor chip was custom-designed in-house to host three reduced graphene oxide electrochemical biosensors and was implemented on a printed circuit board (PCB, manufactured at Eurocircuits NV). The sensor chip was built on a standard 4-layer PCB, 1.6 mm thick, typically consisting of an FR4 core with two copper (Cu) layers on each side, separated by a prepreg insulating layer (fiberglass reinforced with epoxy resin). The outer Cu layers were used to realize the sensor electrodes and connection pads. As shown in Figure 3a, the sensor chip features three distinct detection sites spaced 3 mm apart within a microchannel measuring 1.2 cm in length and 1 mm in width. The microchannel was patterned in the solder mask layer of the PCB (in black, as shown in Figure 3a), typically deposited and patterned to protect the copper surface and to prevent solder shorting between components during assembly. The gold-plated Cu electrodes on the sensor chip were crafted with dimensions of 250 × 220 μm^2^ and a height of 40 μm. Each pair of electrodes maintained a separation distance of 200 μm.

To fabricate the biosensors, GO was drop casted between the electrodes. Subsequently, the chip underwent a heating process at 180 °C for 1 h to effectively reduce GO. More details on the fabrication of rGO biosensors can be found in [27]. To functionalize the biosensors, streptavidin was pipetted over the rGO region at a concentration of 100 µg/mL in 50 mM phosphate buffer, at pH 6.5, and incubated overnight in a humidity chamber. Next, the chips were washed using 50 mM phosphate buffer, at pH 7.4, and distilled H_2_O followed by one-hour incubation in blocking solution (50 mM phosphate buffer, pH 7.4, containing 10 mg/mL BSA). The sensor chip was then rinsed with distilled H_2_O and sealed with a PDMS slab bearing a microchannel, which served as a guiding pathway for analytes to be detected on the biosensors. A schematic representation of the sensor biofunctionalization and assay procedure is depicted in Figure 3b.

For the fabrication of the PDMS sensor microchannel, 12 mm × 1 mm × 0.1 mm, in length, width, and depth, respectively, a mold was fabricated by 3D printing. Then, the microchannel was fabricated from PDMS using replica molding, as described previously in Section 2.2.

**Figure 4:**
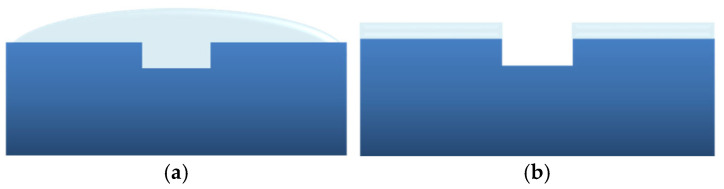
(**a**) PDMS prepolymer drop casted on the sensor microchannel before spin-coating. (**b**) Liquid PDMS interlayer after spin-coating.

In order to ensure a robust seal for the sensor PDMS microchannel on the PCB sensor substrate (Figure 3a), a thin PDMS interlayer was implemented. To this end, liquid PDMS prepolymer (10:1) mixture was applied by drop casting onto the molded microchannel surface, followed by spin-coating at 3500 rpm for 30 s to form a uniformly thick layer on the PDMS surface while eliminating any excess liquid PDMS from within the microchannel (as shown schematically in Figure 4). Subsequently, the PDMS microchannel was aligned and positioned onto the sensor PCB chip, after which the two components, along with the liquid PDMS interlayer, were carefully heated (at 100 °C for 1 h) and fused together to achieve a secure sealing. It was particularly critical to address potential leakage issues arising from the presence of electrical interconnects on the sensor chip surface (Figure 3a), which possessed a height of approximately 40 µm, exceeding the thickness of the liquid PDMS interlayer, which measured around 13 µm. To tackle this challenge, an aluminum chip-holder was employed during the PDMS heating process, ensuring the absence of any undesirable leaks or disruptions of the fluid continuity to the sensors.

To confirm that the PDMS prepolymer did not obstruct the microchannel, measurements of the microchannel depth were performed after its bonding to the sensor using a 3D profilometer (Profilm3D from Filmetrics, San Diego, CA, USA), both before and after the sealing procedure.

To control the flow of solutions in the OoC platform, a peristaltic pump (INSTECH P.625; Instech Laboratories Inc., Plymouth Meeting, PA, USA) was employed. A flow rate of 20 μL/min was applied to introduce the solutions onto the sensor channel and then the flow was reduced to 2 μL/min during the rest of the experiment. Electrical measurements on the sensors were carried out with a HP34401 digital multimeter, which was computer-controlled using the Labview program.

## 3. Results and Discussion

### 3.1. Integrated OoC Platform

**Figure 5:**
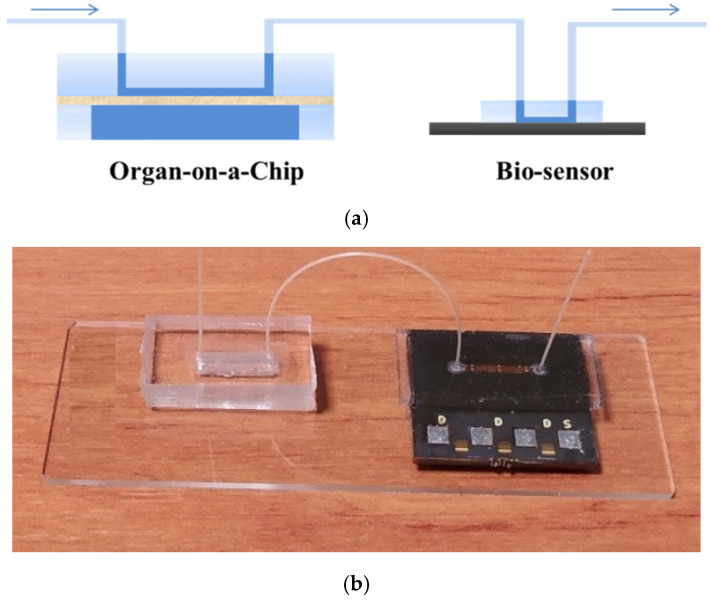
(**a**) Schematic representation of in-series integration of the OoC and the biosensor chip; (**b**) picture of the final layout.

An OoC platform was developed incorporating the microfluidic perfusion chamber and the sensor chip, connected in series with plastic tubing, as illustrated in Figure 5. This arrangement facilitates the flow of the medium through the OoC microchannel, alongside the substances secreted by the cultured cells, directly into the microchannel lying above the biosensor chip. In addition, this arrangement prevents contact or interaction between cells and electrodes, thus mitigating possible biocompatibility risks.

The rGO sensor array chip was sealed with a PDMS microchannel which served as a guiding pathway for the medium to traverse with the analytes through the sensor detection sites. In order to ensure a robust seal for the sensor microchannel, a thin PDMS interlayer was employed to (reversibly) bond the sensor PCB onto the PDMS microchannel, while not obstructing the microchannel. To confirm the latter, measurements of the microchannel depth were performed before and after its bonding to the sensor chip using a 3D optical profilometer. The optical profilometry showed that the height of the uncoated sensor PDMS microchannels was 88.9 ± 6.8 μm, while the thickness of the PDMS prepolymer interlayer applied in the area around the microchannel was measured at 12.86 ± 0.05 μm. Following the bonding and detachment of the microchannel from the sensor chip, the microchannel height was recorded to be 103.8 ± 2.7 μm. The results clearly indicate successful removal of the liquid PDMS prepolymer from the interior of the microchannel, while simultaneously the observed increase in the overall microchannel height can be attributed to the presence of the PDMS interlayer covering only the surface adjacent to and around the microchannel (as shown schematically in Figure 4b). These findings affirm the efficacy of the sealing process and the successful removal of liquid PDMS from within the microchannel and the sensing area.

**Figure 6:**
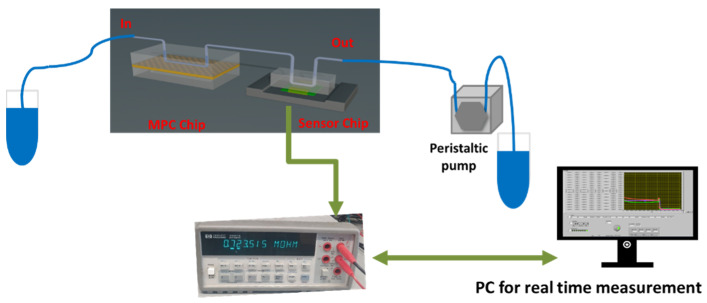
Experimental setup of the OoC platform.

To control the flow of solutions in the OoC platform, a peristaltic pump (INSTECH P625; Instech Laboratories Inc., Plymouth Meeting, PA, USA) was employed and a constant flow rate of 20 μL/min was applied during the experiments to introduce the solutions onto the sensor channel, and then the flow was reduced to 2 μL/min during the rest of the experiment. Electrical measurements on the sensors were carried out with a HP34401 digital multimeter, which was computer-controlled using the Labview program. The experimental setup of the OoC platform is presented schematically in Figure 6.

For the immunoreaction, a biotinylated antibody against IL-6 at a concentration of 2.5 μg/mL in 0.1mM phosphate buffer was then injected, using the peristaltic pump, into the biosensor microchannel for half an hour at a flow rate of about 20 μL/min. Subsequently, a buffer solution of 0.1mM was injected for 10 min followed by different IL-6 concentrations.

### 3.2. In Vitro Cell Culture in the MPC

**Figure 7:**
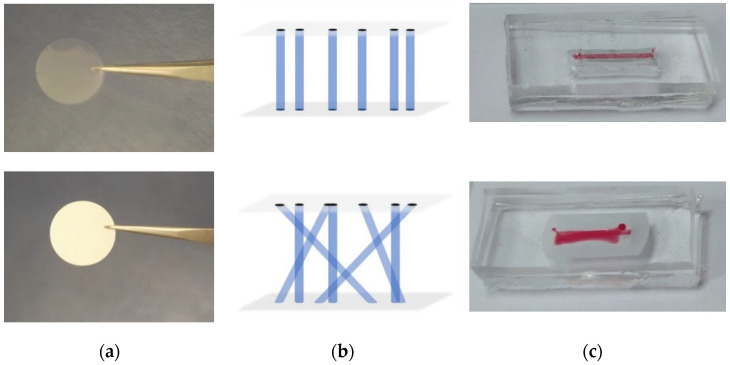
(**a**) Pictures of the membranes. (**b**) Illustration of the inner structure of transparent (**top row**) and semi-transparent (**bottom row**) cell culture membranes. (**c**) Leakage evaluation using colored water in OoCs with hydrophobic transparent (**top row**) and hydrophilic semi-transparent (**bottom row**) cell culture membranes.

The 3D OoC microchannel chip consisted of two PDMS components, one including the microchannel for cell culture, and the other serving as a reservoir for supplying nutrient fluid to the cells separated by a cell culture membrane [28] placed between these PDMS components of the OoC. Two different types of polyethylene terephthalate (PET) membranes of 12 μm thickness and 0.4 μm pore size (ipCELLCULTURE™ track-etched membrane filters) were evaluated as cell culture substrates. The first type included transparent hydrophobic membranes with a low density of parallel pores, enabling cell observation under an optical microscope, while the second one comprised semi-transparent hydrophilic membranes with a high density of crossed pores, which are more suitable for cell mobility experiments (Figure 7a). After OoC chip bonding, when colored water was introduced into the microchannel of the chips with different membrane types, leakage was observed when the semi-transparent membrane was employed, which could be attributed to its hydrophilic nature (Figure 7c). Thus, the transparent hydrophobic membranes were selected for cell culture experiments.

To calculate the OoC microchannel’s resilience to flow, liquid was injected at various flow rates in the microchannel. Transparent membrane chips were used for this purpose, and liquid flow rates from 1 to 1400 μL/min were tested. No leaks were observed at any of the tested flow rates, indicating that the proposed platform can withstand flow rates at least two orders of magnitude higher than typical flow rates used in OoC platforms. Additionally, for the maximum flow rate employed, a pressure drop of 5611 N/m^2^ was estimated.

**Figure 8:**
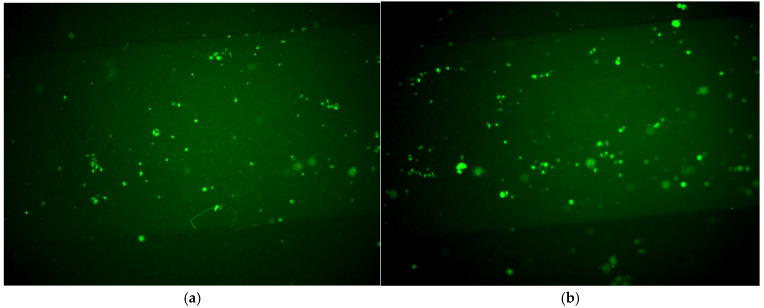
Fluorescence images obtained after (**a**) 24 h and (**b**) 72 h of cell culture into the microchannel.

To assess the viability of cells cultured on the proposed OoC platform, a suspension of 10^6^ cells/mL (200,000 cells/chamber or 65,000 cells/cm^2^) was introduced by means of a pipette onto the culture chamber, and the cells were incubated for 24 and 72 h (Figure 8a,b). The cell capture efficiency of the platform was calculated by determining the number of adherent cells after 24 h of culture through cell counting with appropriate image analysis software, and was found to be 70%. Furthermore, the cells proliferated unbiased in the chamber with a proliferation rate of 520 cells/cm^2^h, calculated by cell counting after 3 days of culture.

Based on the results above, the PMC developed herein was proved efficient for the viability and proliferation of MCF7 (tumor) cells, hosting cultures for 3 days.

### 3.3. IL-6 Detection

The immunochemical detection of IL-6 with the rGO sensors was based on the immobilization of a specific antibody against IL-6 onto the rGO area between the two gold electrodes. Upon the binding of IL-6 to the immobilized antibody, a decrease in the resistance recorded between the two electrodes was expected. However, to be able to detect the interaction between the immobilized antibody and the antigen, the Debye length of electromagnetic field should exceed the thickness of the biomolecular layer formed. As antibodies and proteins are large molecules, in order to achieve the immunochemical detection of a specific protein with the sensor, it was necessary to select immunoreaction buffers of sufficiently low concentration to increase the Debye length [29,30]. More specifically, the dimensions of IL-6 are approximately 1.8 nm, while the height of antibodies is ~10 nm, thus a buffer with a Debye length > 10 nm should be used. Taking into account that phosphate buffer with concentrations of 0.1, 1.0, and 10 mM exhibit a Dedye length of about 21, 6.65, and 2.11 nm, respectively, a 0.1 mM phosphate buffer was selected for the electrical measurements.

**Figure 9:**
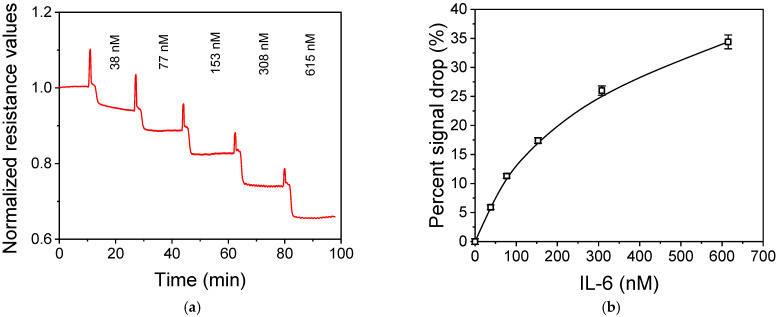
(**a**) Normalized responses obtained from sensors modified with the specific antibody against IL-6 upon sequential flow of IL-6 solutions with concentrations ranging from 38 to 615 nM. The spikes observed at the points prior to each IL-6 solution introduction correspond to the points at which the flow was stopped for the solutions exchange. (**b**) IL-6 calibration curve expressed as percentage drop of the sensor resistance versus the IL-6 concentration. Each point is the mean of 3 measurements ± SD.

To avoid excessive loss of the binding capacity of the specific antibody against IL-6, instead of its direct immobilization by physical adsorption onto the rGO electrodes, an indirect immobilization approach was followed. For this purpose, the antibody was biotinylated and then run over a sensor array modified with streptavidin through physical adsorption. In Figure 9a, the normalized response of sensors modified with streptavidin and biotinylated anti-IL6 antibody upon sequential flow of IL-6 solutions with concentrations ranging from 38 to 615 nM (1.0–15 μg/mL) is provided. As shown, as the IL-6 concentration increased, the resistance drop in the sensor modified with the IL-6 antibody also increased, with a response time ranging from 1.0 to 1.5 min (for a signal drop to 90% of the steady-state resistance value). Based on the cumulative signal drop observed for the different IL-6 calibrators flown over the chip, the calibration curve presented in Figure 9b was obtained. Taking into account that the percent signal variation for the zero calibrator values was 0.5, a detection limit corresponding to +3SD values was calculated at 11.5 nM (0.3 μg/mL).

According to Ortega et al. [8], the IL-6 concentrations excreted in the culture medium after muscle cell electrical stimulation could reach values of about 1 μg/mL, whereas with chemical stimulation the IL-6 values could increase up to 2.5 μg/mL. Thus, although the detection limit achieved here (0.3 μg/mL) does not reach the physiological IL-6 values in human serum (<10 pg/mL), it is sufficient for determining IL-6 excreted by stimulated cells.

**Figure 10:**
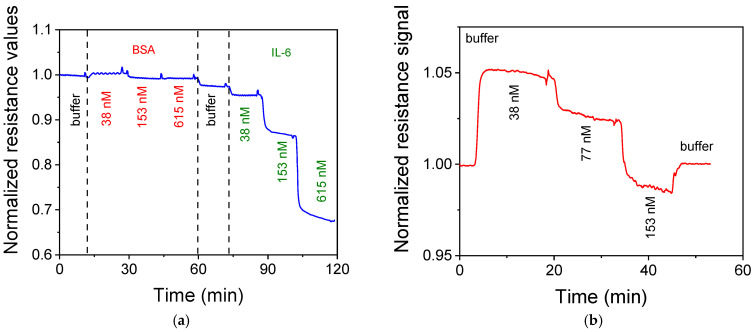
(**a**) Normalized response obtained from a sensor modified with the specific antibody against IL-6 upon sequential flow of BSA and IL-6 solutions with concentrations ranging from 38 to 615 nM. (**b**) Normalized response obtained from a sensor modified with an antibody against PCT upon sequential flow of IL-6 solutions with concentrations ranging from 38 to 153 nM. The spikes observed at the points prior to each solution introduction correspond to the points at which the flow was stopped for the solutions exchange.

To confirm that the responses observed were due to specific interactions between the immobilized sensor antibody and the IL-6 in the solution, the responses of a sensor modified with the specific anti-IL-6 antibody upon the sequential flow, first of BSA and then of IL-6 solutions, with concentrations between 38 and 615 nM, were recorded. As shown in Figure 10a, there was no noticeable response when the BSA solutions were flown over the sensors. On the contrary, a significant response was observed when IL-6 solutions with the same concentration were run over the sensor. Additionally, when a biotinylated antibody against procalcitonin (PCT) was immobilized onto streptavidin-modified sensors instead of the antibody against IL-6, the introduction of the IL-6 solutions caused at the beginning a slight increase in the sensor signal, and then a gradual decrease as the IL-6 concentration increased (Figure 10b) which, however, was considerably lower compared to those obtained for the same IL-6 concentrations, indicating the specificity of the response obtained from the sensors modified with the IL-6-specific antibody.

The monoclonal antibody used in this study to modify the sensors and enable capture of IL-6 is highly specific for the analyte, as verified by the manufacturer. In addition, we tested its specificity towards IL-8, IL-18, and TNF-α using an Enzyme Immunosorbent assay, where the antibody used in this study was employed as the capture antibody. We did not find any detectable cross-reactivity using the cross-reactants under evaluation to concentrations up to 1.5 μg/mL.

The rGO-based immunosensor chip developed herein was shown to be successfully integrated downstream an MPC, providing a fertile OoC platform for the continuous and real-time monitoring of the cell microenvironment. The sensor chip modified with the specific antibody against IL-6 was demonstrated to be sensitive (with a LOD of 0.3 μg/mL) and specific for the detection of IL-6, a protein secreted by cells (renal, tumor, etc.) in response to a variety of stimuli, regulating various events (immune response, inflammation, cell differentiation, survival, and apoptosis). The implementation of OoC components commercially manufactured, such as the biosensor chip developed herein, demonstrates the potential for the mass production and standardization of OoCs. In this proof-of-concept work, in addition to PCB, PDMS was implemented as a laboratory-friendly approach for sealing the sensor PCB chip. As a next step, other, more mass-production-amenable materials and technologies will be chosen, such as adhesive polymeric foils for sealing the sensor chip.

The fact that the estimated LOD for IL-6 is higher than that reported for other sensing chips [16,17] indicates that further work is necessary for the optimization of the sensor’s performance. More specifically, the strategies for sensitivity improvement include the testing of strategies for oriented antibody immobilization, so as to maximize the antibody binding capacity, test antibodies from different sources, and optimize electrode and flow microchannel structures to enhance antibody–antigen interaction. Following optimization, the sensor chip will be tested for the real-time monitoring of IL-6 secreted by cells cultured in the perfusion chamber under chemical stimulation.

The plug-and-play capability of the biosensor developed herein demonstrates its flexibility as it can easily interface with a range of similar sensors, tailored to different biomarkers without redesigning the core electronics or mechanics. In addition, using universal electrical connectors (e.g., electrical pins or PCB mounting) improves scalability, as the same base system can support multiple sensor types with minimal redesign. In addition to IL-6, the detection of other relevant and multiple biomarkers will also be attempted, with different antibodies immobilized on the sensors designed for specific capturing of biomarkers secreted from cells, such as TNF-α. Furthermore, the implementation of PCB-based sensors enables the monitoring of physical parameters such as pH and oxygen, as such sensors implemented on PCB have already been demonstrated and reported [31,32]. Therefore, the herein developed biosensor is modular, adaptable, and capable of supporting the monitoring of diverse extracellular parameters and volume scaling, ultimately lowering the barrier to adoption.

In the future, we also intend to bring the two chips together on the same PCB substrate, thus avoiding external tubing and increasing the degree of integration and the reliability of the OoC platform. Additional PCB-based sensors with other capabilities, such as flow sensing [33], controlled heating [34,35], and other functions indispensable for the operation of an OoC, could then also be implemented on the same PCB-based OoC. Such platforms are envisioned to dramatically improve the speed and quality of drug discovery and disease treatment at low costs. In fact, PCB-based components, such as the herein developed biosensors, play a critical role in enhancing cost efficiency, reproducibility, and the potential for industrial-scale production of biosensors. Cost-efficiency is ensured by manufacturing using well-established, low-cost, and automated fabrication processes and materials like FR-4 or flexible substrates. Reproducibility is also greatly improved, as automated PCB fabrication ensures highly consistent traces, dimensions, and layouts, and reduces batch-to-batch variability in sensor electrical performance and signal quality. In mass production, PCB-based sensors combined with PCB-based microfluidics could reach a fabrication cost close to the cost-effective paper-based disposables [36,37].

## 4. Conclusions

Towards the integration of biosensors into organ-on-a-chip (OoC) platforms, in order to provide real-time monitoring of the cellular microenvironment, we successfully integrated immunobiosensors into a modular OoC platform for the immunochemical detection of a protein biomarker. In particular, IL-6 was used as an example of a mediator protein secreted as part of the immune response in various inflammatory conditions. A rGO-based biosensor array, commercially manufactured on a PCB substrate, was strategically integrated through meticulous sealing downstream the OoC platform, and was tested for the specific detection of IL-6 with a LOD in the nanomolar range. This configuration will allow fluid to flow through the OoC to pass through the cultured cells secreting biomarkers, and traverse the biosensor microchannel, where biomarkers such as IL-6 can be detected in a quantitative manner. In this particular setup, on-chip cell culture images and sensor measurements with reference IL-6 concentrations demonstrated the successful development and functionality of our integrated OoC platform. The developed biosensor successfully detected concentrations of IL-6 up to several tens of nanomolars, demonstrating that this configuration enables the direct and real-time detection of proteins, and can facilitate precise monitoring and analysis of cellular responses in a continuous and dynamic manner. Next, we plan to optimize the sensor for increased sensitivity and integrate the now-separate MPC and sensor modules into a single chip implemented on the same PCB substrate.

## Data Availability

The original contributions presented in this study are included in the article. Further inquiries can be directed to the corresponding author.
